# A Genetic Polymorphism of the Endogenous Opioid Dynorphin Modulates Monetary Reward Anticipation in the Corticostriatal Loop

**DOI:** 10.1371/journal.pone.0089954

**Published:** 2014-02-25

**Authors:** Mikhail Votinov, Juergen Pripfl, Christian Windischberger, Klaudius Kalcher, Alexander Zimprich, Fritz Zimprich, Ewald Moser, Claus Lamm, Uta Sailer

**Affiliations:** 1 Social, Cognitive and Affective Neuroscience Unit, Department of Basic Psychological Research and Research Methods, Faculty of Psychology, University of Vienna, Vienna, Austria; 2 MR Center of Excellence, Center for Medical Physics and Biomedical Engineering, Medical University of Vienna, Vienna, Austria; 3 Department of Neurology, Medical University of Vienna, Vienna, Austria; 4 Department of Psychology, University of Gothenburg, Gothenburg, Sweden; Chiba University Center for Forensic Mental Health, Japan

## Abstract

The dynorphin/κ-opioid receptor (KOP-R) system has been shown to play a role in different types of behavior regulation, including reward-related behavior and drug craving. It has been shown that alleles with 3 or 4 repeats (HH genotype) of the variable nucleotide tandem repeat (68-bp VNTR) functional polymorphism of the prodynorphin (PDYN) gene are associated with higher levels of dynorphin peptides than alleles with 1 or 2 repeats (LL genotype). We used fMRI on N = 71 prescreened healthy participants to investigate the effect of this polymorphism on cerebral activation in the limbic-corticostriatal loop during reward anticipation. Individuals with the HH genotype showed higher activation than those with the LL genotype in the medial orbitofrontal cortex (mOFC) when anticipating a possible monetary reward. In addition, the HH genotype showed stronger functional coupling (as assessed by effective connectivity analyses) of mOFC with VMPFC, subgenual anterior cingulate cortex, and ventral striatum during reward anticipation. This hints at a larger sensitivity for upcoming rewards in individuals with the HH genotype, resulting in a higher motivation to attain these rewards. These findings provide first evidence in humans that the PDYN polymorphism modulates neural processes associated with the anticipation of rewards, which ultimately may help to explain differences between genotypes with respect to addiction and drug abuse.

## Introduction

A growing number of genetic neuroimaging studies suggest that functional polymorphisms in genes regulating the dopamine system play a crucial role in mediating reward-related behavior [1], [2], [3], [4]. Among these genes, prodynorphin (PDYN), the gene coding for the dynorphin opioid peptides, is a strong candidate for influencing a range of neuronal circuits, including the reward pathways. Dynorphins bind with highest affinity to kappa opioid receptors (KOP-R), but also to mu and delta opioid receptors [5], [6], [7]. Moreover, dynorphin-like peptides and their receptors (i.e., the kappa-opioid receptor) affect dopamine release in the striatum and prefrontal cortex. More specifically, dynorphin inhibits the release of dopamine and is therefore assumed to play a critical role in the negative feedback regulation of dopamine [8], [9], [10], [11].

In the human brain the PDYN gene is predominately expressed in the medial prefrontal cortex, the amygdala, the dentate gyrus and the striatum [12], [13]. While several functions have been associated with the striatum, the medial orbitofrontal cortex (mOFC) and the amygdala, it is widely accepted that they play a dominant role in processes related to reward anticipation and consumption, in the control of mood, and motivation, as well as in stimulus-response learning [14], [15], [16], [17], [18], [19]. In line with these functions, there is evidence that increases in PDYN neurotransmission may contribute to the pathogenesis of depression, anxiety-like behavior, dysphoria, and drug addiction [20], [21], [22], [23].

We have previously [24] identified a functional genetic polymorphism that seems to be particularly relevant in this respect. This polymorphism is located in the PDYN gene promoter region with one to four repeats of a 68-bp element containing one binding site per repeat for the transcription factor AP-1 (c-Fos/c-Jun). It was observed that alleles with 3 or 4 instances of this variable nucleotide tandem repeat (68-bp VNTR) are associated with higher levels of mRNA, and thus, higher levels of dynorphin peptides and higher degrees of dopamine inhibition, in comparison to alleles with only 1 or 2 repeats. Alleles with 3 or 4 repeats have been referred to as “high” (H) expression alleles, and those with 1 or 2 as “low” (L) expression alleles [24].

It is still a matter of debate how this particular polymorphism relates to reward processing and its dysfunction in addiction. While several studies observed that individuals with a higher number of alleles (and hence higher dynorphin levels) showed increased susceptibility to the development of cocaine [25], methamphetamine [22] and heroin [21] dependency, others did not find such a relationship between genotype and drug abuse [26], [27]. Numerous psychopharmacological studies indicate, though, that chronic drug abuse as well as KOPr agonists enhance the expression of dynorphin, lead to adaptations in KOPr’s second-messenger signaling in the mesolimbic reward system, and therefore are able to influence and regulate the brain’s reward system (for review, see [28]).

Despite its high expression in limbic areas [12], [13], it is still unclear how PDYN modulates the function of the mesolimbic corticostriatal loop and affects reward-related behavior in healthy human participants on a systems level. To address this issue, we used functional magnetic resonance imaging (fMRI) to assess neural processes particularly related to the anticipation of monetary rewards and losses in participants showing differences in the prodynorphin 68 bp VNTR polymorphism. To this end, we used a well-established experimental task, the monetary incentive delay (MID) task [29]. This task is known to reliably trigger robust activation in the ventral striatum (nucleus accumbens (NAc)), the amygdala, and in medial prefrontal cortex, and activation strength in these areas provide a measure of an individual’s sensitivity to anticipated reward [30], [31], [32], [33]. We focused on anticipatory neural processes in our analyses. This focus was based on the rationale that by experimental design of the task, trials for the anticipation of gains and losses are balanced. Hence, we expected that a focus in anticipatory processes would give us better access to test brain activity in response to cues with different valences and magnitudes. Moreover, the task timing would have made it difficult to statistically separate processes specifically related to behavioral outcomes (gains and losses) vs. their anticipation. Notably, these areas were our primary regions of interest (a) because of their high level of PDYN gene expression, (b) because they are all part of the dopamine system, and (c) because they are all considered part of the limbic-corticostriatal motivational system [34]. We therefore expected that neural responses in these areas during the anticipation of a reward would be mediated by dynorphin, and assessed this hypothesis by investigating activation differences between individuals with high or low expression of PDYN.

## Methods

### Participants

In a random sample of healthy Europeans, the frequency of H-homozygotes is 50%, and L-homozygotes is about 10% [35]. Based on this distribution, we screened 286 healthy Caucasian volunteers with no history of psychiatric or neurological disorders or contraindications for MRI scanning for their genotype in the PDYN 68-bp VNTR. This skewed natural distribution was deliberately not replicated in the current study. Rather than assessing a representative sample, our aim was to compare neural and behavioral differences between genotype groups. For reasons of robustness and validity of the statistical analyses, we therefore decided to recruit three genotype groups of about equal group size. All participants provided written informed consent and the study protocol was approved by the ethics committee of the Medical University of Vienna. Based on genotyping, all volunteers were classified to one out of three groups with high (HH), intermediate (HL or LH; hitherto LH for simplicity), or low (LL) PDYN expression. The distribution of genotypes in the screening sample was: HH –142 participants, LH –113, LL –31. 25 participants from each group (total N = 75) were invited to the fMRI experiment, which was performed in a double blind fashion. The HH and LH groups participating in the fMRI experiment were matched for age, gender, alcohol and tobacco use (based on the average number of cigarettes and alcoholic drinks consumed a day) with the LL group. Due to lack of compliance or technical problems, data of four participants were excluded from the analyses. The final sample included 23 participants in the LL group, 23 in the LH group, and 25 in the HH group. The gender (female/male) distribution across groups was 13/10 in the LL group, 13/10 in the LH group, and 14/11 in the HH group (mean age, for LL 24.3±7.2; for LH 23.09±4.1; for HH 23.28±4.41; differences not significant). The minimum age of participants was 19 and maximum age was 40. All participants were drug free, as determined by a drug test (Dip-Test MULTI 5/1, Dipro med, Austria) testing for opiate, amphetamine and cannabinoid substance use, applied prior to scanning.

Participants filled in the BIS/BAS scale questionnaire measure [36] which assesses sensitivity of the behavioral inhibition and approach systems (i.e., sensitivity to reward and punishment). Whereas the BIS controls risk assessment and avoidance behaviors in response to threat, the BAS is thought to control appetitive behaviors in response to reward. Participants received a participation fee as well as additional payments according to their performance in the task.

### Genetic Analyses

DNA was determined based on saliva samples collected using a self-collection kit designed for the collection and storage of DNA (Oragene DNA, DNA Genotek, Ottawa, Canada). A commercial kit (Qiagen, Hilden, Germany) was used for DNA extraction. PDYN genotyping was performed according to established procedures at the DNA laboratory of the Department of Neurology of the Medical University of Vienna. In short, purified DNA was diluted into a PCR reaction mix consisting of 20 mM Tris-HCl (pH 8.8), 50 mM KCl, 1.5 mM MgCl2, deoxynucleotide triphosphates each at 0.4 mM, 10 pmol of each primer, and 0.6 U of Taq polymerase in a total volume of 30 µl. Amplification conditions were 30 s at 94°C, 45 s at 62°C, and 45 s at 72°C for 30 cycles using the following primers, which flank the entire promoter region: upstream (P1), 5′-AGC AAT CAG AGG TTG AAG TTG GCA GC; and downstream (P2), 5′-GCA CCA GGC GGT TAG GTA GAG TTG TC. The amplification products were resolved on a 2.5% agarose gel stained with ethidium bromide.

### Task Design

**Figure 1:**
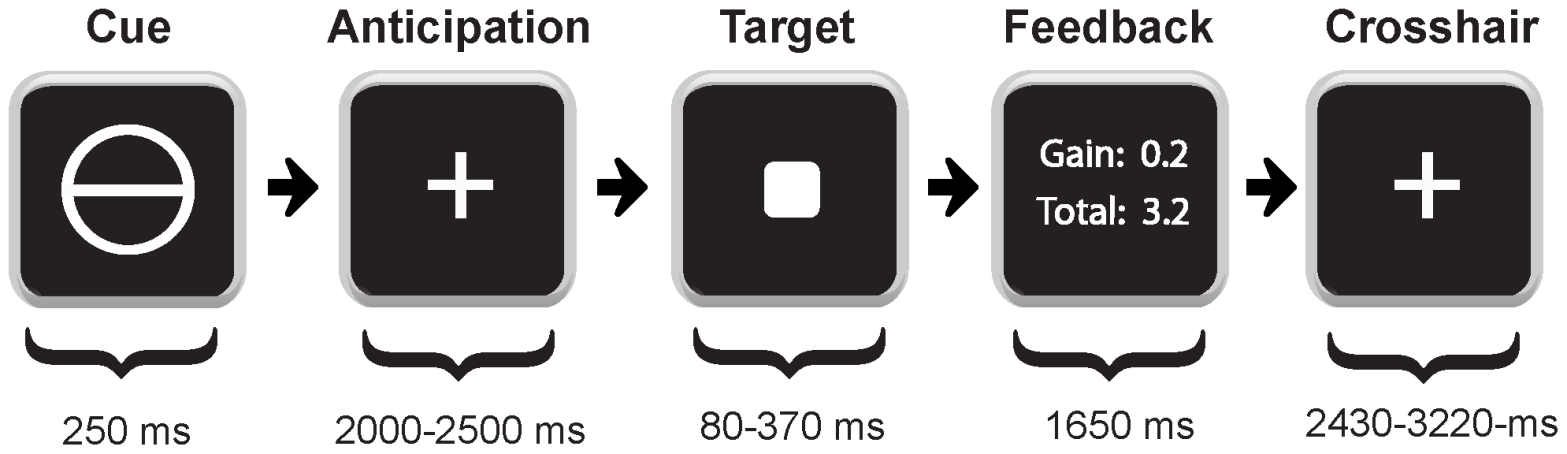
Schematic illustration of one trial of the monetary incentive delay (MID) task performed by subjects in the MRI scanner.

All participants, before entering the scanner, had taken part in ten practice trials to familiarize them with the task and to minimize learning effects during the experiment. To be sure that the participants had understood the task, they were then asked to explain the task’s rules to the experimenter. The Monetary Incentive Delay (MID) task [29] (see Fig. 1) consisted of two runs, with 72 trials in each run. During each trial, participants saw one of seven geometrical cues for 250 ms. Next, they waited for a target square to the appearance of which they had to respond with a button press as fast as possible. During target anticipation, a fixation crosshair was shown, and the anticipation period was varied randomly between 2000–2500 ms. Feedback was presented for 1650 ms immediately after disappearance of the target, informing participants about whether they had won or lost money during that trial. In addition, their cumulative score of all trials was displayed. Cues represented potential reward (indicated by a circle), potential punishment (indicated by a square), or a control condition with no monetary outcome (indicated by a triangle). “Monetary Gain” cues signaled the possibility of winning € 0.20 (a circle with one horizontal line), € 1 (a circle with two horizontal lines), or € 3 (a circle with three horizontal lines). Similarly, “Monetary Loss” cues signaled the possibility of losing € −0.20 (a square with one horizontal line), € −1 (a square with two horizontal lines), or € −3 (a square with three horizontal lines). Cues representing “no monetary outcome” (€ 0) were denoted by a triangle. Trial types were pseudorandomly ordered within each run.

Participants were instructed to press the response button as fast as possible upon presentation of the target cue in order to win or to avoid losing money. The display duration of the target cue was varied (80–370 ms) to ensure that participants would be able to respond in time in 2/3 of all trial types. Reaction time data were analyzed in SPSS 20.0 (SPSS Inc., Armonk, USA) using two separate repeated-measures ANOVAs. One ANOVA had two levels for the valence of Possible Outcomes (gain = positive, loss = negative) and three levels for Magnitude (€ 0.2, € 1, € 3) as within-subjects factors, and Genotype (groups LL, LH, HH) as a between-subjects factor. The other ANOVA had three levels of the factor Possible Outcome (positive, calculated as mean RT across the three gain cue magnitudes; negative, mean RT across the three loss cue magnitudes; and neutral) as a within-subject factor, and Genotype (LL, LH, HH) as a between-subjects factor. The rationale for the latter analysis was to compare RT in the incentivized conditions (where either winning money, or avoiding its loss was possible) to the neutral condition. If the sphericity assumption was violated (significant results in Mauchly’s test of sphericity), degrees of freedom were corrected using Greenhouse-Geisser estimates of sphericity. Significance was evaluated at P<0.05. Post-hoc tests with Bonferroni correction for multiple comparisons were applied. All data are reported as means ± SD.

### MRI Scanning

MRI scanning was conducted on a 3 Tesla TIM Trio whole body scanner (Siemens, Germany). Participants were scanned using the manufacturer’s 32-channel head coil. Functional images were obtained with a single-shot echo planar imaging (EPI) sequence. The image acquisition parameters were as follows: repetition time (TR) = 1.8 s, echo time (TE) = 38 ms, flip angle (FA) = 90°, 294 whole-brain volumes (matrix size 128×128, FoV = 190×190 mm^2^, 3 mm slice thickness). For anatomical registration, we obtained high-resolution 3D T1 anatomical images after the fMRI runs (magnetization prepared rapid gradient echo sequence, TR = 2.3 s, TE = 4.21 ms, 1.1 mm slice thickness, 900 ms inversion time, 9° flip angle).

Image analysis was performed using the SPM8 software package (www.fil.ion.ucl.ac.uk/spm) implemented in MATLAB (Mathworks Inc., Natick, USA). Preprocessing included correction for slice-timing differences [37], realignment to the first image to adjust for movement, segmentation, normalization to standard MNI space (at isotropic voxel size), and smoothing with a Gaussian filter (8 mm). The first level (individual subject) analyses were set up using the general linear model approach, with events of interest being modeled by regressors.

The anticipation-related responses were modeled for all seven incentive cues: possible gain €0.20, €1, €3, neutral €0, possible loss € −0.20, € −1, € −3. The two types of feedback (win or loss) and target cue were also modeled. Contrast images of these regressors from the first level were then entered into second level random-effects analyses to allow for group-level inference, and in particular to test for differences between the three groups with different PDYN genotypes.

The contrasts we assessed focused on neural activation differences during the anticipation conditions, namely Gain Anticipation>Neutral Anticipation (GA>NA), Loss Anticipation>Neutral Anticipation (LA>NA), and Gain Anticipation>Loss Anticipation (GA>LA).

### Anatomical ROI Analysis

The main objective of our study was to test specific hypotheses derived from the literature on the role of dynorphin on reward related neural activation (see Introduction above). We therefore specifically assessed possible group differences in left and right amygdala, left and right ventral striatum, and medial orbitofrontal cortex (mOFC). These analyses were performed using a regions-of-interest (ROI) analysis approach, using independent anatomical ROIs [38].

For the amygdala and mOFC, image masks for these ROIs were defined in standard stereotactic space, using the anatomical AAL atlas [39] templates provided in Marsbar toolbox (http://marsbar.sourceforge.net/). In accordance with the method proposed in Plichta et al (2012) [40], the mask for the ventral striatum was defined by a conjunction of the “caudate head” template provided in the WFU-PickAtlas (Version 3.3, Wake Forest University, School of Medicine, Winston-Salem, North Carolina; www.ansir.wfubmc.edu) and the “accumbens” template taken from the Harvard–Oxford Subcortical Structural Atlas. Mean parameter estimates within these five masks were extracted for all gain and loss anticipation cue contrasts, against the implicitly modeled fixation baseline. This approach was preferred over contrasting gain and loss cues against neutral cues for two related reasons. First, it enabled us to analyze each magnitude (€ 0.20, € 1, € 3, € −0.20, € −1, € −3) separately. Second, in such an analysis, the unequal numbers of monetary (18 trials per cue magnitude) and non-monetary cues (36 trials for the neutral condition) could have been problematic.

Group and condition differences, and their interactions, were analyzed in SPSS 20.0 (SPSS Inc., Armonk, USA) using a Linear Mixed Model (LMM) with restricted maximum likelihood estimation [41], [42]. In this model, we evaluated the within-subjects fixed factors ROIs (factor levels: left and right VS, left and right amygdala, mOFC), cue valence (factor levels: gain and loss), cue magnitude (factor levels: € 0.20, € 1, € 3), the between-subjects fixed factor genotype groups (factor levels: LL, LH, HH), and the random factor subjects in a full-factorial fixed-effects model. Schwarz’s Bayesian criteria [43] were used to determine the best-fitting variance-covariance structure, which was determined to be diagonal. Bonferroni corrected post hoc comparisons were used to examine interactions and omnibus main effects. Significance was evaluated at P<0.05. All data are reported as means ± SD.

### Whole-brain Analyses

This is the first investigation of the role of genetic variation related to dynorphin in reward processing. Hence, to take into account the exploratory character of our study, we not only tested specific hypotheses using the ROI approach, but also performed group comparisons using whole-brain analyses to explore possible group differences outside the *a priori* ROIs. These analyses were implemented in SPM8 using an ANOVA model with the between-subject factor group (LL, LH, HH).

In addition, we tested for activation irrespective of group membership to determine whether the MID task as implemented here yielded activation patterns similar to previous reports. The statistical threshold of these analyses was set to P = 0.05, corrected for multiple comparisons at the voxel-level using random Gaussian field theory (as implemented in SPM8).

### Effective Connectivity Analysis

In addition to the ROI functional segregation and whole-brain analyses, we explored whether the groups also differed with respect to effective connectivity between mOFC (which was the only area showing robust functional segregation group differences, see Results) and other parts of the reward network. These analyses were implemented within the framework of psychophysiological interaction (PPI) analyses [44]. Due to the exploratory character of this analysis and because activation differences were highest between HH and LL, we assessed differences between these two extremes only. The seed region we used for the PPI analysis was contained within the anatomical mOFC ROI used for the functional segregation ROI analyses, and it was centered on the maximum of a cluster within the mOFC ROI that had been revealed when exploring the whole brain analysis at P = 0.001, k = 100 voxels extent threshold (uncorrected). Using this cluster’s maximum as the center of the PPI seed, instead of using the whole mOFC ROI, was based on the rationale that this subregion of mOFC showed the strongest response in the functional segregation analysis, and therefore would most likely also yield more sensitive results for the PPI analysis.

The seed region of the PPI analysis was based on a sphere with 10 mm diameter in anterior mOFC with MNI coordinates (0 60 −10), with the center defined by the peak of the group difference (HH>LL) of the contrast GA>NA (gain anticipation vs. neutral anticipation). Using this sphere, we calculated the psychophysiological interaction term as the product of the mean time course in this region and the respective psychological variable. The psychological variable was gain anticipation, defined as the contrast GA vs. NA. All three variables (time course in seed region, psychological variable, and interaction term) were entered into a new general linear model for each subject. On the second level, an ANOVA with the between-subject factor group (genotypes HH vs. LL) and the within-subject factor run (1st and 2nd run) was set up. Linear contrasts (with factor run pooled) were performed to compare the parameter estimates of the psychophysiological interaction term between groups. The statistical threshold of these analyses was set to P<0.05 (corrected for multiple comparisons on the cluster-level, cluster selection intensity threshold P = 0.005).

### Correlation Analysis

Additionally we performed a correlation analysis (Pearson) between extracted parameter estimates from all ROIs and scores from the BIS/BAS score and the total number of repeats in the alleles (from 2 to 8). While the former analysis served to identify possible associations between trait personality differences and neural activation, the latter analysis aimed to assess in a more fine-grained manner than the analyses based on group categorization whether there is a linear relationship between genetic variation and neural responses. To compare correlation coefficients, we converted them to z-scores which were then compared using a Fisher’s test for two independent correlations.

## Results

### Behavioral Data

The allelic distribution of the genotype of interest PDYN (LL, LH, HH) was in Hardy-Weinberg equilibrium, χ^2^ (2) = 1.39, p = 0.5. This indicates that the screened population was genetically homogeneous for the distribution of the PDYN genotype.

In the ANOVA assessing valence and magnitude, there was no significant main effect of genotype on reaction time (F (1, 2) = 0.75, p = 0.47, η^2^ = 0.022), and no significant interaction with Genotype as a factor (all p-values >0.55).

However, the repeated measures ANOVA comparing the positive, negative and neutral outcome conditions revealed a main effect of Potential Outcome (F (1, 1.57) = 184, p<0.001, η^2^ = 0.73). Again, though, no significant effects including the factor Genotype were found (all p-values >0.1). Post-hoc tests for pooled RT data for all subjects demonstrated that mean RT for “monetary gain” cues (200±17.21) was significantly faster than RT for “monetary loss” cues (204±18.5, p = 0.005) and mean RT for both “monetary gain” and “monetary loss” cues were significantly faster than RT for “no monetary outcome” cues (229.5±19.6, for all p<0.001).

Similarly, the BIS/BAS scale, which in two subscales assesses sensitivity of the behavioral inhibition and approach systems (i.e., sensitivity to reward and punishment), did not reveal significant differences between groups (BIS: p = 0.5/BAS: p = 0.49): HH – BIS = 2.85±0.63/BAS = 3.10±0.4; LH BIS = 2.89±0.49/BAS = 3.16±0.41; LL– BIS = 3.03±0.38/BAS = 3.23±0.32). Analyses of the subscales BAS drive, BAS fun seeking and BAS reward responsiveness did also not reveal any significant differences between groups (all p-values >0.5).

### fMRI Data for All Subjects

**Figure 2:**
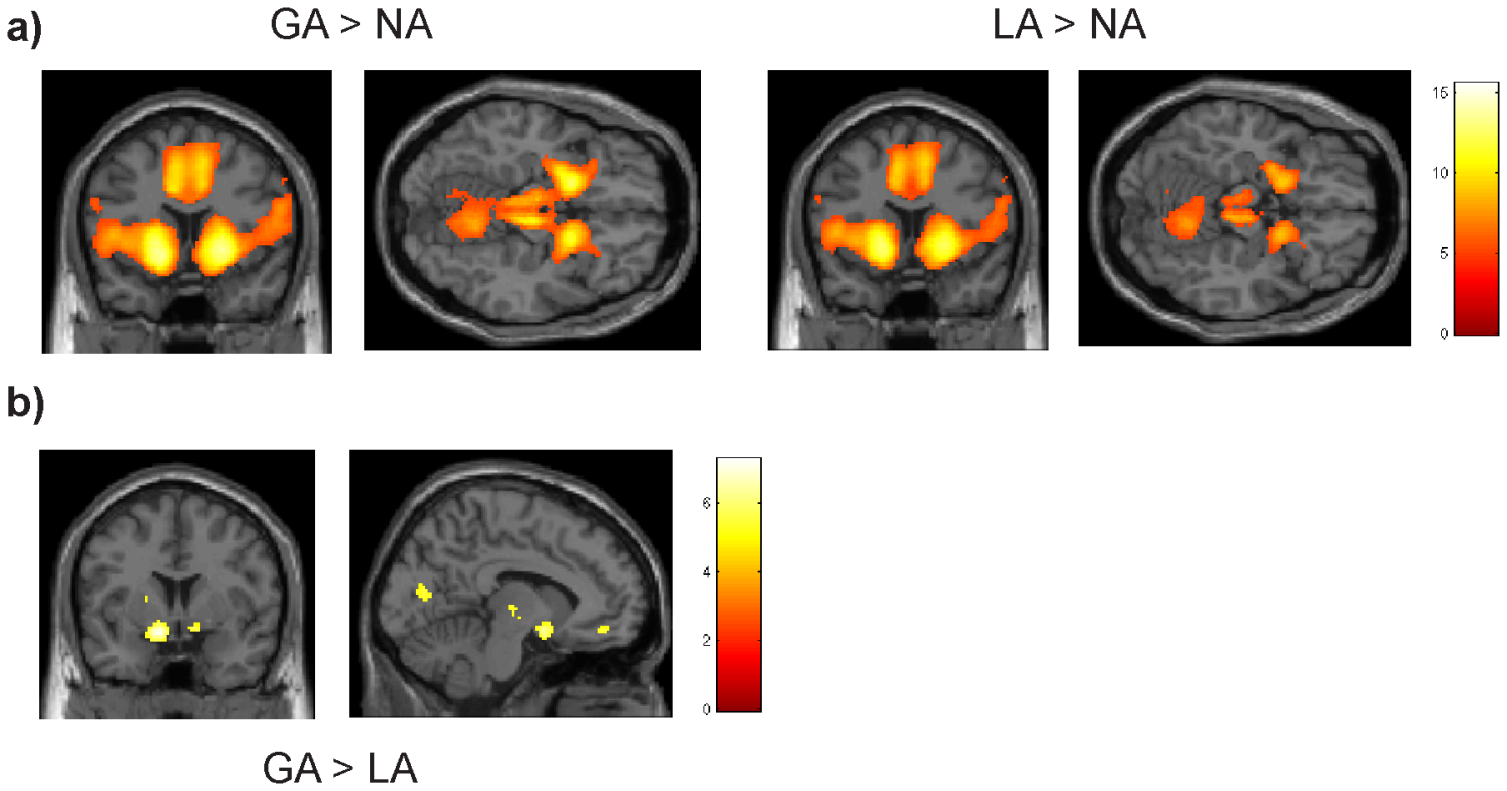
Whole brain activation of all 71 participants for reward and loss anticipation conditions. (**a**) The comparison between gain and neutral anticipation (GA>NA) conditions and between loss and neutral anticipation (GA>LA) conditions showed activation in bilateral striatum, amygdala, insula, midbrain, anterior cingulate cortex and cerebellum in both incentive conditions relative to the neutral no outcome control condition. (**b**) The contrast between gain anticipation and loss anticipation (GA>LA) revealed activation in ventral striatum, ventro-medial prefrontal cortex VMPFC, thalamus and posterior cortex (PC). The threshold is p = 0.05, corrected for multiple comparisons on the voxel level (see methods).

The first fMRI analyses primarily aimed to determine whether the activation patterns we observed across groups were in line with previous studies on reward anticipation. Consistent with previous reports [29], [30], [33], the MID task revealed greater activation for gain as well as loss anticipation, compared to the neutral control condition (contrasts Gain Anticipation vs. Neutral Anticipation (GA>NA); Loss Anticipation vs. Neutral Anticipation (LA>NA)), in bilateral striatum, insula, amygdala, anterior cingulate cortex (ACC), thalamus, and midbrain (Fig. 2 a). Also in line with previous findings, the contrast GA>LA revealed higher activation during gain anticipation in bilateral striatum and ventro-medial prefrontal cortex (VMPFC) (Fig. 2 b). No activated clusters were revealed by the reverse contrast (LA>GA).

### Effect of Genotype on Activation during Reward Anticipation - ROI Analysis

Having ascertained that the experimental task we used robustly and in line with previous findings activated cortico-limbic regions of interest associated with reward processing, we assessed the modulation of these activations by the PDYN polymorphism.

The LMM analysis did not reveal a significant main effect for Genotype F(2,69.745) = 2.07, p = 0.13 (HH = 2.55±5.5, LH = 1.349±4.2, LL 1.03±3.44), but significant interactions Genotype×Valence F(2,1030) = 4.91, p = 0.007, and Genotype×ROIs F(8, 550) = 8.3, p<0.001. The other interactions did not reach significance (all p’s >0.46).

**Figure 3:**
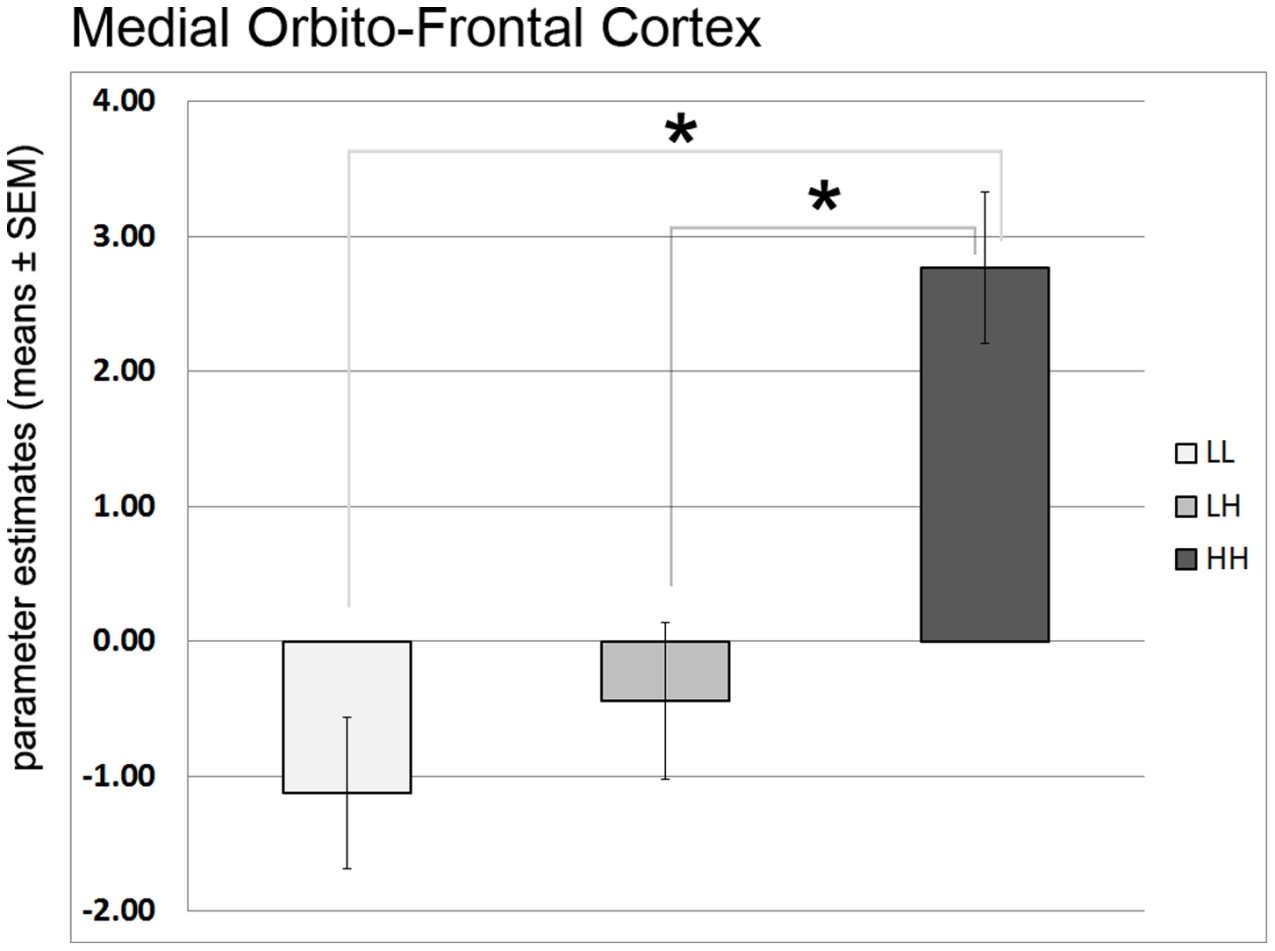
BOLD signal parameter estimates from anatomical regions during gain anticipation in different (68-bp VNTR) prodynorphin promoter polymorphism genotypes (LL-, LH-, or HH-alleles). The HH (“high level pDYN expression”) group shows significantly higher activation (see *) in the mOFC compared to the LL (“low level pDYN expression”) and LH groups.

To scrutinize the significant LMM interactions, we performed Bonferroni corrected post-hoc comparisons. These comparisons demonstrated significant differences (Fig. 3) only during gain anticipation, where the HH group showed higher activation than the LL group (p = 0.003) and the LH group (p = 0.019; HH: 2.77±8; LH: −0.43±6.5, LL: −1.12±4) in mOFC. In addition, explicitly testing for a linear trend in activation increases across the three groups yielded a significant effect (p = 0.049), with the highest activation in HH, intermediate activation in LH, and lowest activation in LL.

While we did not observe significant differences in ROIs other than mOFC, testing for a linear trend in activation increases yielded a significant result for the left amygdala as well, with highest activation in HH, intermediate activation in LH, and lowest activation in LL (p = 0.029; HH = 2.34±3.2; LH = 1.99±2.4; LL = 0.58±2.3). Interestingly, and unexpectedly, neither significant differences between groups nor a linear trend were found for the ventral striatum.

### Effect of Genotype on Activation during Reward Anticipation - Whole-brain Analysis

Exploratory whole-brain analyses did not reveal any additional areas outside the ROIs, even when lowering the threshold to p = 0.001 (uncorrected).

### Effective Connectivity

**Figure 4:**
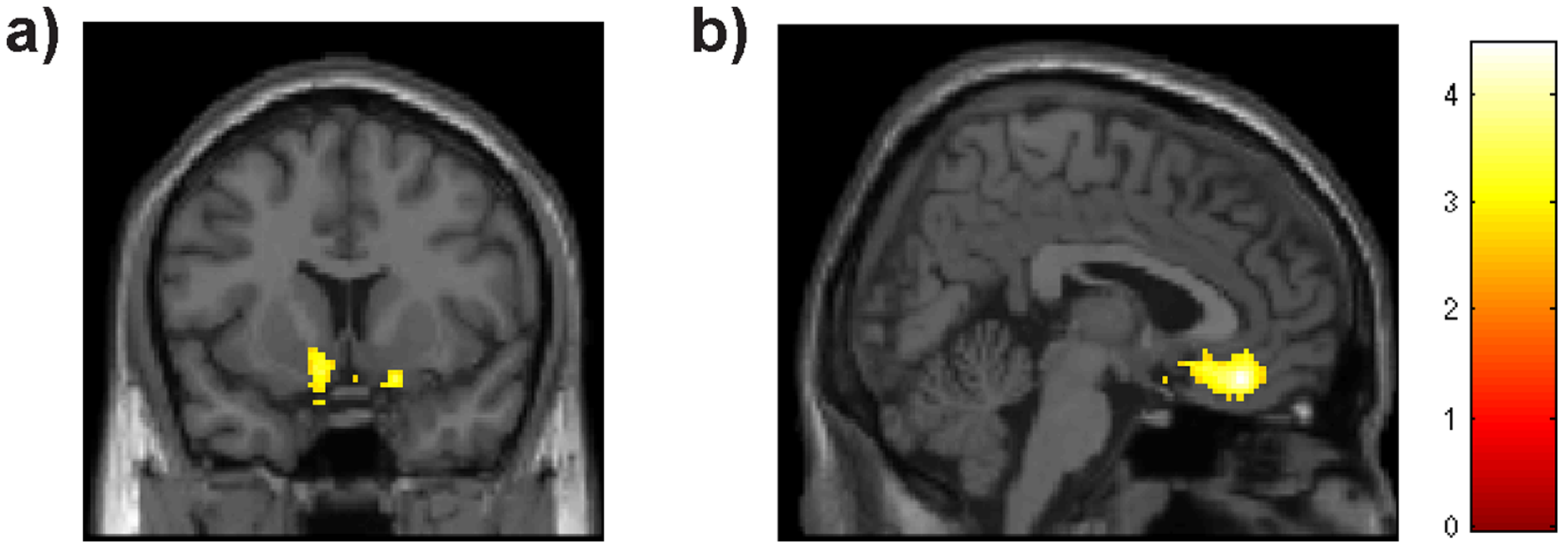
The PPI analysis during gain anticipation shows higher functional coupling of the anterior mOFC seed region of the HH group with the ventral striatum (a), subgenual anterior cingulate cortex and VMPFC (b) regions compared to the LL genotype group. The threshold is p = 0.05 cluster level corrected for multiple comparisons (see methods).

The PPI analysis investigated which areas showed higher functional coupling with the anterior mOFC seed region, during gain anticipation (recall that the main finding of the functional segregation analyses was a group difference in mOFC during gain anticipation). The HH group showed higher PPI than the LL group in the left and right ventral striatum (peak coordinates −8/10/−14; T = 3.43 18/10/−16; T = 3.47), subgenual ACC/VMPFC, (4/36/−16; T = 4.45), and the ventrolateral PFC (−18/32/−24; T = 4.27) (Fig. 4 a,b; p = 0.05 corrected at cluster level, cluster selection intensity threshold p = 0.005). During loss anticipation, the HH group showed higher functional coupling of anterior mOFC with the VMPFC −6/20/−22; T = 3.92 than the LL group (only at a threshold of p<0.001 uncorrected, though). The reverse contrasts (LL>HH, for gain and loss anticipation) did not reveal any significant clusters.

### Correlations with Total Allele Number and BIS/BAS Score

Correlating the total number of repeats in the alleles (from 2 to 8) and activation in ROIs during gain and loss anticipation revealed significant positive correlations in the mOFC (r = 0.214, p = 0.036) and the left amygdala (r = 0.218, p = 0.034) during gain anticipation only.

ROI activation during gain anticipation in the mOFC was negatively correlated with the BAS Drive subscore in the LL group (r = −0.46, p = 0.012), while the LH (r = 0.03, p = 0.43) and the HH (r = 0.2, p = 0.16) groups showed no correlations. Importantly, comparison of the correlation coefficients revealed a significant difference between the HH and LL groups only (Z =  −2.27, p = 0.01). No significant correlations were observed between any of the other ROIs activation during gain and loss anticipation and BAS subscales scores, or the BIS score.

Additional exploratory Pearson correlation analysis between mean reaction time for gain cues and the extracted activation from mOFC during gain anticipation for all 3 groups demonstrated a significantly negative correlation (r = −0.34, p = 0.001). In addition, we explored whether the three genotype groups showed significantly different correlations, which was not the case (all p-values >0.15, correlation coefficients ranging from −0.15 to −0.44).

Note that virtually identical correlation results were obtained when contrasting gain activation (pooled across the three magnitude levels) against activation during anticipation of the neutral cue. This implies that the correlation results also hold when using a higher level control condition.

## Discussion

Our findings provide first evidence that neural activity during reward anticipation is modulated by the PDYN (68-bp VNTR) genetic polymorphism. Individuals with the HH genotype, associated with higher levels of mRNA coding for dynorphin peptides, showed highest activation in medial orbitofrontal cortex during gain anticipation compared to carriers of the LH and LL genotype. In addition, the HH group showed stronger functional coupling of this area with VMPFC/sACC and the ventral striatum during reward anticipation. Furthermore, the LL group showed a significant negative correlation of mOFC activation with a questionnaire measuring approach behavior and sensitivity to rewards, while the HH group did not. Importantly, the correlation coefficients for the two groups differed significantly. Additionally, the correlation between mean reaction time for gain cues and the extracted activation from mOFC during gain anticipation demonstrated a significantly negative correlation for the HH and LH groups, but not for the LL group.

Taken together, these results point towards a larger sensitivity for upcoming rewards in individuals with the HH polymorphism.

As expected, the MID task engaged a brain network previously reported in numerous similar studies [29], [30], [33]. The whole brain analysis of all participants revealed activation in the ventral striatum, midbrain, bilateral insula, amygdala and anterior cingulate cortex. These results confirm that our ROIs were involved in the processing of reward anticipation. The reaction time analysis also revealed that, in general, participants responded faster to cues with monetary gains and losses, than for cues with no monetary outcome, which is in line with previously published data [33], [45], [46], [47]. However, previous study has documented behavioral differences using a similar task setup, and even smaller sample sizes [47]. This study has compared healthy controls to clinical group such as pathological gamblers, though, while we compared only neurotypical healthy participants. Future studies with larger sample sizes are needed to replicate our findings, as well as to clarify whether the lack of differences in behavioral results is due to sample size limitations, or other factors.

The genotype group comparison of ROI data revealed larger activation in the mOFC during gain anticipation in the HH genotype than in both the LL and the LH/HL genotypes. Notably, activation in mOFC during gain anticipation showed a linear increase across the three genotype groups, and so did activation in the left amygdala. This finding was further corroborated by the correlation analysis, which showed that the number of repeats in alleles was significantly and positively correlated with activation in mOFC and amygdala. In a more fine-grained manner than the analyses based on a categorization of individuals into groups, these analyses therefore suggest a linear relationship between PDYN genotype (and hence available dynorphin) and activation in key areas related to reward and motivation.

Additionally, the correlation between mean reaction time for gain cues and the extracted activation from mOFC during gain anticipation demonstrated a significantly negative correlation in the full sample and no genotype group differences. One therefore might speculate that this indicates that the neural processes in mOFC during gain anticipation are generally related to reward sensitivity (in all three genotype groups), as this might decrease response times. Future studies using more direct measures of the latter concept are needed, though, to test this hypothesis more directly.

Numerous animal and human studies have already established that medial OFC and amygdala are involved in reward processing, and that this role seems especially pronounced during the anticipation of rewards [48], [49], [50], [51], [52], [53], [54], [55]. For example, visual food stimuli were found to cause activity in the amygdala, medial frontal/orbitofrontal cortex before the meal (i.e., the reward anticipation phase), but not after the meal (i.e., during reward delivery) [56]. Another food study demonstrated that trait reward sensitivity was correlated with activations to images of appetizing foods in a neural network, including amygdala, ventral striatum, midbrain and orbitofrontal cortex [57]. It has been suggested that the amygdala encodes the motivational significance of cues and that OFC uses this information in the selection and execution of an appropriate behavioral strategy [49]. Via its connection to the amygdala, the OFC is also involved in attaching emotional valence to an upcoming reward [58].

This suggests that the monetary cues were more salient for HH participants than for LH and LL participants. Thus, PDYN seems to alter processing of information about expected rewards. This might also explain its role in addiction [23], which according to recent concepts is a disorder in reward anticipation and incentive salience (“wanting”) rather than hedonic “liking” [59]. However, this salience interpretation will need to be clarified by future studies, as for methodological reasons we did not ask participants to deliver saliency or affect ratings.

In addition to the larger overall activation in the mOFC, there was also a larger coupling of the mOFC with VMPFC and the ventral striatum during reward anticipation in the HH genotype. This result is consistent with findings from a connectivity-based parcellation study of the human orbitofrontal cortex that reported a functional connection between the medial OFC cluster, the medial PFC, the PCC, the ventral striatum and the lateral prefrontal cortex [60]. Since it has been suggested that both the ventral striatum and the VMPFC form the core of a “valuation system” [61] and provide information about the prevailing expectation of reinforcement, the higher connectivity in the HH group during gain anticipation is in line with our interpretation that this group shows an increased expectation and/or an altogether higher sensitivity for upcoming rewards [62].

Surprisingly, we did not observe any difference between groups in the ventral striatum. This may be explained by findings from a study by Margolis et al. (2006) which demonstrated that KOP-R agonists inhibit VTA dopamine neurons that project to the medial prefrontal cortex, but not those that project to the nucleus accumbens. Additionally, the level of medial prefrontal cortical dopamine level was decreased after intra-VTA KOP-R agonist perfusion in that study [8]. This might explain the difference in brain activity between HH and LL groups in the mOFC and the absence of a difference in ventral striatum. Additionally, the alterations in dopamine release to the prefrontal cortex may also contribute to changes in the functioning of this region and changes in functional connectivity of mOFC with other brain regions. It is likely that alterations in PDYN expression may affect the glutamate projection in ventral striatum [63] and in the mOFC.

The present study is limited by the lack of studies about basal dopamine dynamics for healthy participants with the PDYN (68-bp VNTR) gene polymorphism. Therefore, we are currently not able to prove whether the observed changes are due to a direct effect of PDYN, due to a modulation of dopaminergic transmission, or due to interaction with glutamatergic transmission. Moreover, the possible homeostatic adaptation of dopamine release in response to higher levels of dynorphin peptides in the HH group should also be considered. For example, Chefer et al (2005) demonstrated that loss of KOP-R and the resulting disinhibition of dopamine neurons triggers short- and long-term dopamine transporter adaptations that maintain normal dopamine levels, despite enhanced dynorphin release [64]. It could well be that the same neuroadaptation takes place in the HH group, leading to an increase of dopamine release which could be the cause for the increased activation levels. However, since the present study was not designed to answer this question, future investigations are needed to assess how PDYN genotype and dopamine interact during reward processing. Another potential limitation of this study is the small sample size of the three genotype groups and, possibly as a consequence, the lack of significant differences in the behavior of the genotype groups, despite differences in the respective correlation between brain activation and the personality questionnaires. This may be due to behavioral data having smaller power, which requires more subjects to detect an effect. In contrast, brain activation as an intermediate link between genes and behavior may reveal changes in brain activation that are not manifested in behavior [65]. However, previous study has documented behavioral differences using a similar task setup, and even smaller sample sizes [47]. This study has compared healthy controls to clinical groups such as pathological gamblers, though, while here we compared only neurotypical healthy participants. Future studies with larger sample sizes are therefore needed to replicate our findings, as well as to clarify whether the lack of differences in behavioral results is due to sample size limitations, or other factors. In conclusion, the group-independent findings of anticipation related activity in the ventral striatum, amygdala and orbitofrontal cortex are in line with numerous animal and human brain studies indicating that these areas play a prominent role in reward expectation. The fact that OFC and left amygdala were most activated in the HH group might characterize higher reward sensitivity and motivation in participants with higher PDYN levels. Furthermore, the HH group had stronger functional connectivity with the ventral striatum and VMPFC. Overall, this could provide a hint for understanding the possible vulnerability of this group to reward (or drug) seeking behavior. In fact, it has been suggested that the OFC may play a role in the drug craving [66], and that genotypic variation of PDYN is associated with substance abuse and addiction [23]. It has been proposed that the effect of dynorphins on reward processing and addictive behavior might be mediated by stress [67]. Thus, the differences between genotype groups observed in the present study may also be due to a different involvement of the stress axis.

Taken together, our results are a first step towards clarifying a genetically determined link between the PDYN (68-bp VNTR) polymorphism and neural activity in the limbic corticostriatal loop.
